# Design, Development, and Testing of a Device for Gene Electrotransfer to Skin Cells In Vivo

**DOI:** 10.3390/pharmaceutics14091826

**Published:** 2022-08-30

**Authors:** Aleksandra Cvetkoska, Janja Dermol-Černe, Damijan Miklavčič, Simona Kranjc Brezar, Boštjan Markelc, Gregor Serša, Matej Reberšek

**Affiliations:** 1 Faculty of Electrical Engineering, University of Ljubljana, Tržaška 25, 1000 Ljubljana, Slovenia; 2 Institute of Oncology Ljubljana, Department of Experimental Oncology, Zaloška 2, 1000 Ljubljana, Slovenia

**Keywords:** electroporation, gene electrotransfer (GET), plasmid DNA, pulse generator, pulse delivery protocol

## Abstract

Gene electrotransfer (GET) is considered one of the most efficient, safe, reproducible, and cost-effective methods of gene therapy, in which a gene is delivered to the cells in the form of a plasmid DNA vector by a method known as electroporation. To achieve successful electroporation, cells must be exposed to sufficiently high electric fields generated by short-duration, high-voltage electrical pulses that result in a temporary increase in plasma membrane permeability. The electrical pulses are generated by pulse generators (electroporators) and delivered to the cells via electrodes (applicators). However, there is a lack of standardized pulse delivery protocols as well as certified clinical pulse generators and applicators for gene delivery. In this paper, the development of a new pulse generator, applicator, and pulse delivery protocol for GET to skin cells is presented. A numerical model of electroporated skin developed and tested for two electrode configurations and two different pulse delivery protocols is also presented. An alternative pulse delivery protocol was proposed. The developed pulse generator, applicator, and the proposed pulse delivery protocol were then used in vivo for GET to skin cells in mice. The results showed high efficiency of the proposed pulse delivery protocol for the purpose of GET in mouse skin cells. Specifically, electroporation with the developed pulse generator, applicator, and proposed pulse delivery protocol resulted in higher gene expression in skin cells compared to the currently used pulse generator, applicator, and pulse delivery protocol.

## 1. Introduction

Gene therapy is one of the new and promising therapeutic approaches for the treatment of cancer, in which plasmid DNA vectors containing therapeutic genes are introduced into target cells to induce a therapeutic effect [1]. Gene delivery methods are divided into viral and non-viral methods based on the vectors that carry the information DNA [2]. Gene electrotransfer (GET), a non-viral delivery method, is considered one of the most efficient, safe, reproducible, and cost-effective methods [3,4]. GET allows the genetic material to be delivered directly into tissues (skin, muscle, or tumor) by a method known as electroporation [5,6,7,8]. To achieve successful electroporation, cells must be exposed to sufficiently high electric fields, which leads to a temporary increase in the permeability of the plasma membrane. Electroporation pulses are electrical pulses generated by pulse generators, also known as electroporators, and delivered to the cells (in the tissue) via electrodes (applicator) [9,10] as a necessary accessory part but separate medical device. The pulse parameters are usually set by an operator via a user interface. Electroporation has been shown to significantly increase the efficiency of DNA drug delivery [11]. Therefore, transdermal or intradermal GET is one of the most promising and widely used applications of skin electroporation [12]. However, the translation of skin electroporation into the clinic has been slow and lags behind in vitro and in vivo studies [13,14]. One of the possible reasons is inadequate dosimetry, which impedes comparison of the pulse generators, applicators, and pulse parameters [15]. There is a lack of certified clinical pulse generators and applicators for gene delivery, as well as standardized pulse delivery protocols to enable translation to human applications. Many pulse generators currently in use do not meet their technical specifications and do not verify the delivered waveforms [16]. Different pulse parameters are used with varying success, which renders comparison between the results difficult. The equipment and pulse parameters are often inadequately reported, making the studies not comparable or reproducible. We believe that with adequate dosimetry, predictive modeling, and development of high-quality electroporation devices, the efficiency of skin electroporation treatments can be increased, allowing comparison between treatments and facilitating the translation into the clinics. 

In this paper, we present the development of the new pulse generator, applicator, and pulse delivery protocol for GET to skin cells based on predictive modeling. First, we explain the developed numerical model of the electroporated skin, which allowed testing of different electrode configurations and pulse delivery protocols to achieve the best possible effect for gene delivery. Then, all the necessary requirements and recommendations for simpler design and development of a pulse generator for clinical use are listed and the treatment protocol is suggested. Based on the basic requirements for a medical device, we thus describe the design of the newly developed device for GET to skin cells. We also present the design of the newly developed applicator (noninvasive electrodes) for safe and easy delivery of the electrical pulses. Finally, we present the results of the performed in vivo study in mice and compare our results with the currently used pulse generator, applicator, and pulse delivery protocol.

## 2. Materials and Methods

### 2.1. Numerical Determination of the Optimal Electrode Configuration

Optimal electrode configuration and pulse delivery protocol for gene electrotransfer (GET) were determined numerically. The development of the optimal electrode configuration was based on two criteria:

1. Minimizing collateral damage by minimizing the volume of irreversible electroporation.
2. Maximizing gene transfer efficiency by maximizing the reversibly electroporated volume.

We developed a numerical model of skin, which allowed us to easily test different electrode configurations and pulse delivery protocols. Our skin model was based on multiscale analysis and was constructed according to [17,18], with sensitivity analysis performed as in [15]. The model consisted of eight different layers, also considering anisotropy of tissue conductivity. The electrical conductivity was a function of the electric field described by sigmoid, and the process of electroporation was modeled stationary and sequentially [19]. The thickness of the layers, their initial electrical conductivities, and the threshold values for the maximum increase in conductivity due to electroporation were based on [17,19,20] and are listed in Table 1. 

**Figure 1:**
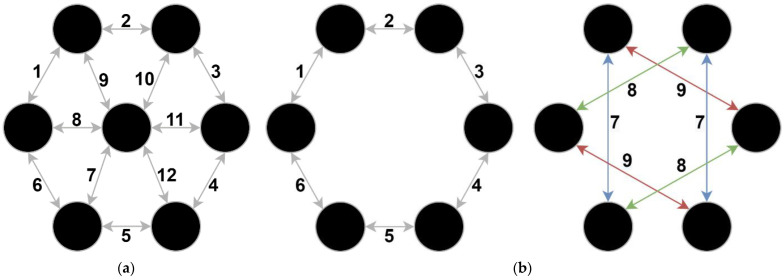
The order of pulse delivery for (**a**) the classical pulse delivery protocol and (**b**) the proposed alternative pulse delivery protocol. The numbers indicate the order of pulse delivery. The arrows indicate the direction of the applied pulse (anode -> cathode).

Based on the previous knowledge, we selected and compared two different pulse delivery protocols: the classical [21] (Figure 1a) and the proposed alternative protocol, similar to [22,23], with addition of pulsing around the perimeter (Figure 1b). The main difference between the applicators, i.e., electrode configurations and pulse delivery protocols, is that the proposed alternative protocol does not include a central electrode. The arrows in Figure 1 indicate which electrodes are used and the order in which the pulses are delivered. In the classical protocol, the pulses are delivered between all adjacent electrodes, first in one direction and then in the opposite direction, i.e., with reversed polarity. In the proposed alternative protocol, the pulses are delivered first between adjacent electrodes on the rim, again switching polarity (Figure 1b, left). To compensate for the missing central pin, the pulses are then delivered between two opposite pairs of electrodes and with the polarity also switched (Figure 1b, right).

### 2.2. Requirements and Recommendations to Be Considered When Designing a Clinical Electroporator for Gene Electrotransfer to Skin Cells

#### 2.2.1. Medical Device Regulation and Standards

A clinical pulse generator (electroporator) for GET to skin cells is considered a medical device for which patient and operator safety must be ensured under both normal and single-fault conditions. In addition, such a device must comply with medical device standards and meet the requirements of local medical regulations, e.g., Medical Device Regulation (MDR) 2017/745 in Europe or CFR (Code of Federal Regulations) Title 21 in the United States (US), in order to be sold on the market, e.g., certification mark (CE) in Europe or FDA (Food and Drug Administration) approval in the USA. 

A clinical electroporator is classified as a Class IIa active therapeutic device, type BF (Body Floating). All technical documentation required for certification of the device should then be based on the established level of risk, i.e., classification class. The main standard to be considered when designing such a device is EN/IEC 60601-1: Medical electrical equipment—Part 1: General requirements for basic safety and essential performance. This standard is a generally accepted criterion for medical electrical equipment, and compliance with this standard has become the main requirement for placing the medical electrical equipment on the market. According to the standard, the essential safety factors that should be considered in the design of the device are: limitation of voltage, current, and energy, limitation of leakage currents, adequate insulation according to the device class, and maintaining safe operation, quality, and efficiency even in the event of a single-fault condition. Electromagnetic compatibility requirements should be met according to the EN/IEC 60601-1-2 standard, while risk analysis should be performed according to the ISO 14971 standard. Other standards that should be considered in the development of a clinical electroporator for GET to skin cells are ISO 13485 for the quality management system, EN/IEC 60601-1-6 and ISO 62366 for usability, ISO 62304 and IEC 80002-1 for medical device software, and IEC 62311 for a battery-powered pulse generator [24].

#### 2.2.2. User and Technical Recommendations

Portability of the pulse generator, i.e., that can be easily transported from one place to another (between different clinics or operating rooms), is often desired by operators. To enable/facilitate portability, the pulse generator must be battery-powered with a rechargeable battery. A battery level indicator is required to allow the operator to estimate the remaining operating time of the device. A pedal control or button on the applicator is necessary to arm and deliver the electrical pulses, so that the operator (clinician) can independently hold the applicator in the sterile field. A touchscreen is preferred (over keyboard and mouse) as the user interface to set pulse parameters manually or automatically (based on the treatment plan). The device should be able to generate the pulse parameters set by the operator (amplitude, pulse duration, pulse repetition rate, etc.) and be equipped with appropriate visual and audible alarm systems to alert the operator to low-risk or high-risk processes or events. Validation of the current and voltage of the output pulses is essential, as is the storage of treatment data for post-treatment analysis and quality control. The device needs to be designed in a way to allow easy maintenance and cleaning. Noninvasive, reusable electrodes must be made of biocompatible material (e.g., medical grade stainless steel), and designed to allow appropriate and safe cleaning. 

The user and technical recommendations were determined based on the operator’s needs and previous user experience with other pulse generators.

#### 2.2.3. Recommended Treatment Protocol for Safe and Efficient Gene Electrotransfer to Skin Cells 

It is recommended that the entire procedure is performed in one room, usually an examination room (in hospitals/clinics). The patient must be informed in advance that some contractions of the underlying muscle are to be expected, but that a local anesthetic should protect against pain, as the penetration of the electric field is not great. The appropriate amount of local anesthetic and plasmid DNA dose to be administered to the patient must be prepared [25]. The device must be in good working order, the battery charged before use, and the applicator must be connected to the device. Once the device is set and ready, the operator can select the pulse parameters. Experienced medical personnel should then perform the local injection of the plasmid DNA. The waiting time between the injection and the application of the electrical pulses is proposed to be between 30 s and 2 min [25]. While holding the handle of the applicator with one hand and lifting the area from the underlying muscle with the other hand (when possible), the operator should start the application of the electrical pulses using a control pedal connected to the device or by pressing the button on the applicator. Monitoring the delivered pulses is important to verify that the voltage and current delivered are consistent with the values set by the operator. After the treatment, the electrodes must be removed and discarded (single-use electrodes) or sterilized for the next use (reusable electrodes). The device then needs to be switched off and cleaned for the next use. If warnings and alarms occur during the treatment, they must not be ignored. In the case of suspicion or malfunction, the manufacturer must be contacted.

### 2.3. System Design

**Figure 2:**
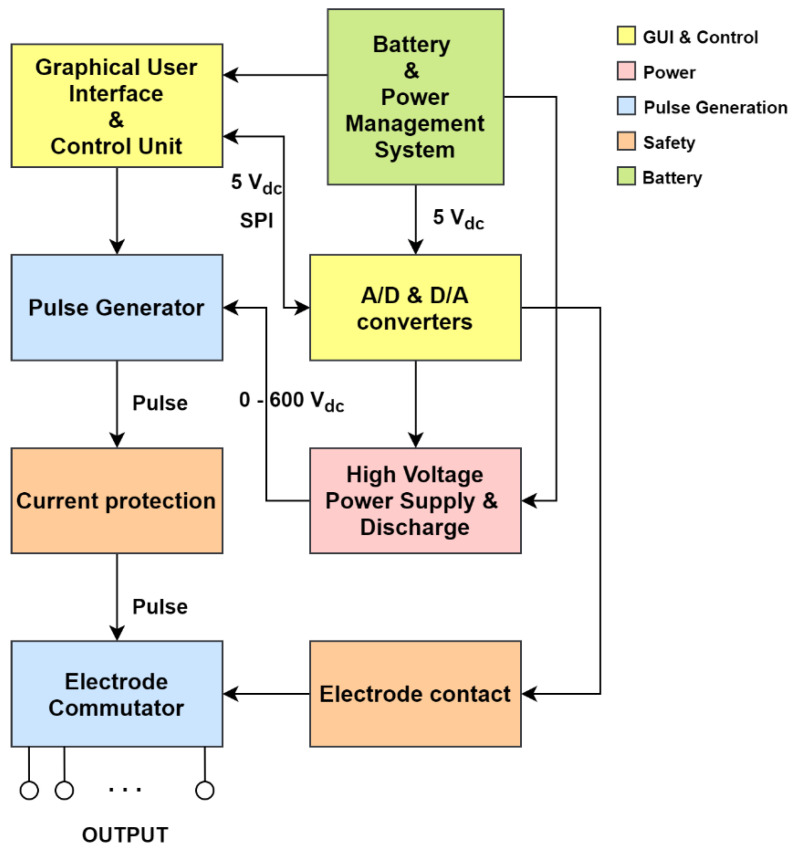
Block diagram of the device for GET to skin cells.

Figure 2 shows a block diagram of the system design for the device for GET to skin cells. Five different functional units were defined prior to development, colored differently depending on the task being performed: Graphical User Interface (GUI) and Control (yellow), Safety (orange), Pulse Generation (blue), Power (red), and Battery (green).

#### 2.3.1. Graphical User Interface (GUI) and Control Unit

**Figure 3:**
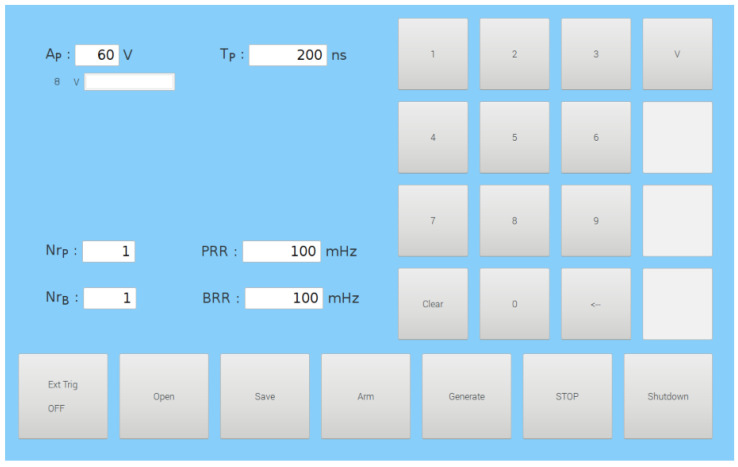
Graphical User Interface (GUI) of the device, displayed on the SunFounder 10.1″ touchscreen. The values of the parameters can be entered using the keyboard on the right side. A_P_—amplitude of the pulse; T_P_—duration of one pulse; Nr_P_—total number of pulses, Nr_B_—total number of bursts; PRR—Pulse Repetition Rate; BRR—Burst Repetition Rate.

The GUI and Control unit (yellow, Figure 2) consists of a graphical user interface (GUI) and an isolation and conversion circuit with Analog-to-Digital (A/D) and Digital-to-Analog (D/A) converters. The GUI of the device was developed on a Raspberry Pi 3 (Raspberry Pi Foundation, United Kingdom) with a LogiPi FPGA circuit installed (Valent Fx, France), which was used as a control unit of the device. The parameters of the electrical pulses are entered into the device through GUI (Figure 3) developed using the GTK3 library (Genome Foundation, USA). The GUI is displayed on a SunFounder 10.1″ 1280 × 800HDMI Touchscreen (Shenzen Headquartes, China). 

The isolation and conversion circuit with A/D and D/A converters provides galvanic isolation of the control signals by optocouplers and enables control of the high-voltage (HV) power supply. The isolation ensures that the high voltage does not transfer to the low voltage part of the device in case of a fault in the high voltage part. The conversion part of the circuit enables the digital control signal to be converted to an analog signal and the voltage to be measured with the A/D converter using the standardized SPI (Serial Peripheral Interface) protocol.

#### 2.3.2. Power

The Power unit (red, Figure 2) provides the power to the device. The high-voltage (HV) power supply consists of an HV DC-DC converter HRL3024S600P (XP Power, Kunshan, China), three HV capacitors B32774D0705K000 (7 µF, 1.1 kV; EPCOS, TDK Corporation, Tokyo, Japan) connected in parallel, and an HV fuse at the output 0090.0004 (4 A, 1 kVdc; Schurter, Lucerne, Switzerland). Thus, the HV power supply has a total capacitance of 21 µF, power of 30 W, and enables controlled power supply from 0 to 600 V.

#### 2.3.3. Pulse Generation

The pulse generation unit (blue, Figure 2) is responsible for generating the electrical pulses and providing the correct voltage supply to the electrodes. The generator has three control inputs (pulse, stop, and discharge), low voltage and high voltage power inputs, and an output for electrical pulses. The pulse control signal can be used to raise the output to 560 V and lower it back to 0 V in 1–5 µs. The stop signal must always be present, otherwise the HV pulse will be turned off in less than 1 µs. The discharge control signal is used to discharge the HV capacitors. 

The electrode switching circuit, i.e., the electrode commutator, switches the electrical pulses between the electrodes according to the selected pulse delivery protocol (Figure 1). The electrode commutator provides the output voltage of the generator to the electrodes in the correct sequence and transmits the output voltage to each individual electrode at the required moment according to the signals from the control unit. We used 14 HE24-1A83 reed relays (Standex, Salem, NH, USA), which can commutate the electroporation output signal to up to seven independent electrodes.

#### 2.3.4. Safety

The safety unit (orange, Figure 2) provides protection in the case of overcurrent at the output of the device and verifies the electrical parameters of the generated HV pulses. The current limiter prevents the current and power from becoming too high when discharging the HV capacitors. In the event of high currents, which may occasionally occur during therapy, the current limiter does not stop the therapy, but only limits it to the maximum expected value of the current during therapy. This allows the therapy to proceed normally even if the current occasionally increases.

In addition, we developed a circuit to check the contact of the electrodes with the skin before delivering the electrical pulses, as this may increase the probability of successful delivery of the pulses. The skin electrode contact detector is designed to distinguish between three different impedance ranges between the electrodes to determine if the electrodes are in contact with the skin, i.e., if they have the appropriate impedance for the pulse generator. The first impedance range that can be determined by the circuit is too low impedance for the generator (no skin contact). The third impedance range that can be determined by the circuit is too high impedance between the electrodes and the skin (too high conductivity range). The impedance range between the first and the third range is the impedance range where the electrodes are in contact with the skin.

#### 2.3.5. Battery 

In order to have a rechargeable device that can be easily transported between different examination rooms, a battery unit (green, Figure 2) was added to the device. It consists of a 24 V battery power supply with a battery power management system. A level indicator was implemented to allow the operator to predict the remaining operating time of the device. For the power supply, six lithium cells 1850CA (BIPOWER Corp., Monterey Park, CA, USA) connected in series with an average voltage of 3.75 V were used. To monitor the performance of the battery system, the MAX17263 integrated circuit (Maxim Integrated, San Jose, CA, USA) was used. The battery management system was implemented on the MAX17263GEVKIT# development board (Maxim Integrated, USA). 

### 2.4. In Vivo Experiments

#### 2.4.1. Plasmid DNA

Plasmid pEGFP-N1 (Clontech Laboratories Inc., Mountain View, CA, USA), encoding enhanced green fluorescent protein (GFP), was prepared from Escherichia coli cultures using the Qiagen Endo-Free Plasmid Mega kit (Qiagen, Hilden, Germany) according to the manufacturer’s instructions and diluted to a working concentration of 1 µg/µL. Plasmid concentration was determined using the Qubit DNA Broad Range kit (TFS, Waltham, MA, USA) using fluorometric quantification with the Qubit 4 Fluorometer (TFS, Waltham, MA, USA). Plasmid quality was assessed using the 260/280 nm ratio determined using the Epoch Microplate Spectrophotometer (BioTek, Bad Friedrichshall, Germany).

#### 2.4.2. Mice

Female 10–12 week-old Balb/c (BALB/cAnNCrl) mice (Charles River Laboratories (Italy)) were used in the experiments. Mice were kept in a specific pathogen-free environment with a 12 h light-dark cycle at 20–24 °C and relative humidity of 55% ± 10%; food and water were provided ad libitum. The experiments were approved by the Ministry of Agriculture, Forestry and Food of the Republic of Slovenia (permission no. 34401-1/2015/43 and U34401-3/2022/11). The experimental procedures were performed in accordance with the guidelines for animal experiments of the EU directive (2010/63/EU) and ARRIVE guidelines.

#### 2.4.3. In Vivo Gene Electrotransfer

Before in vivo gene electrotransfer (GET) of pEGFP-N1 both flanks of mice were shaved and depilated with hair removal cream (Vitaskin, Krka d.d). Considering randomization, each flank was assigned to a different experimental group. Before GET, mice were anesthetized with Isoflurane (Piramal Healthcare UK Limited, London, UK). A 29 G insulin grade syringe (CHIRANA T. Injecta, Stará Turá, Slovakia) was used to inject 25 µL of pEGFP-N1 intradermally at a concentration of 1 µg/µL. Immediately, i.e., within 30 s to 2 min after plasmid injection, the electrical pulses were applied. Two different pulse delivery protocols were used for plasmid delivery. The first was the low-voltage (LV) pulse delivery protocol with an amplitude-to-distance ratio of 170 V/cm (12 pulses, amplitude 60 V, duration 150 ms, pulse repetition rate 2.82 Hz) applied with the Cliniporator (IGEA s.r.l., Carpi, IT) through noninvasive multi-electrode array (MEA, Iskra Medical, Podnart, SI) consisting of six spring-loaded pins arranged in a hexagonal mesh (Figure 1b, first picture with only 6 arrangements) and spaced 3.5 mm apart, as this was found to be optimal [26,27]. The second was the proposed alternative pulse delivery protocol (18 sequences (Figure 1b) with 4 pulses, burst repetition rate 50 Hz, amplitude 560 V, duration of each pulse 100 µs, pulse repetition rate 5 kHz) applied with the pulse generation unit (Section 2.3, Figure 2) and the applicator described in Section 3.2.1 During GET a conductive gel (Gel G006 ECO, FIAB, Vicchio, Italy) was used at the point of contact between the electrodes and the skin to ensure good conductivity. The currents reached with the first pulse delivery protocol were 100 mA, while the currents reached with the second (proposed) alternative pulse delivery protocol were 500 mA.

#### 2.4.4. Image Acquisition and Analysis

To determine in vivo transfection efficiency, mice were imaged with a fluorescence stereomicroscope (excitation: 470/40 nm, emission: 525/50 nm, SteREOLumar V.12, Carl Zeiss, Jena, Germany), equipped with an AxioCam MRc5 digital camera (Carl Zeiss), on days 1, 3, 5, and 7 after GET. The images were subsequently analyzed using FIJI [28]. On each image the transfected area was separated from the non-transfected area by determining the number of pixels with the intensity above the same threshold pixel intensity. From the determined transfected area, the mean fluorescence intensity of the pixels and the integrated density (product of area and mean fluorescence intensity) were determined.

## 3. Results

### 3.1. Numerical Determination of the Optimal Electrode Configuration

**Figure 4:**
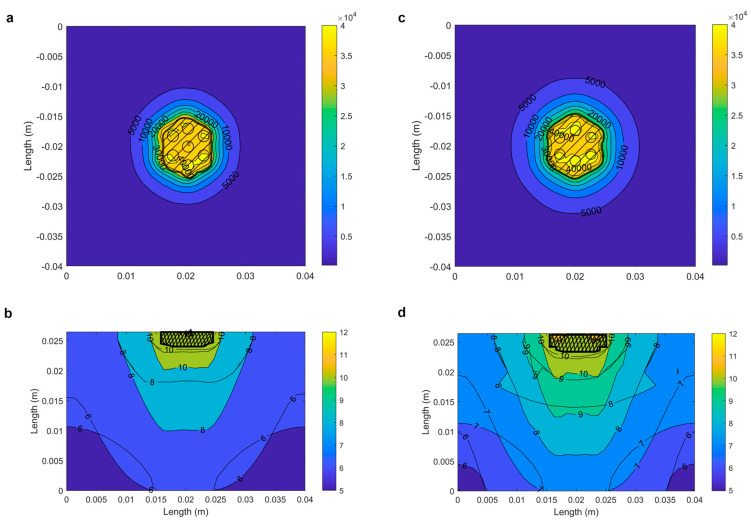
Electric field distribution for (**a**) the classical and (**c**) the proposed alternative pulse delivery protocol (V/m) 2 mm below the skin surface in the hypodermis, where the cells important for the immune response are located. The location of the electrodes is marked with circles. Side view of the natural logarithm of the electric field distribution (V/m) in the middle between the electrodes for (**b**) the classical and (**d**) the proposed alternative pulse delivery protocol. The shaded area shows the area of reversible electroporation. We chose the logarithmic representation as the electric field values differ for ranges and the differences would not be clearly seen otherwise.

The modeling results show that the proposed alternative protocol yields an 8% larger reversibly electroporated volume which also penetrates deeper in comparison to the classical protocol. In addition, by avoiding the use of a central electrode, the damage by irreversible electroporation is reduced by 15% in the proposed alternative protocol compared to the classical protocol. The irreversible damage is mostly concentrated in the stratum corneum directly under the electrodes. The proposed alternative protocol is thus more successful in achieving deeper and more homogeneous reversible electroporated volume than the classical protocol, while collateral damage remains low, suggesting that gene electrotransfer should be more successful with the proposed alternative pulse delivery protocol than with the classical pulse delivery protocol. The electric field distribution and reversible electroporated volume of the classical and proposed alternative protocols 2 mm below the skin surface and as a side view between the electrodes are shown in Figure 4 (a and b for the classical, and c and d for the proposed alternative pulse delivery protocol).

### 3.2. System Design

#### 3.2.1. Applicator—Electrode Development

**Figure 5:**
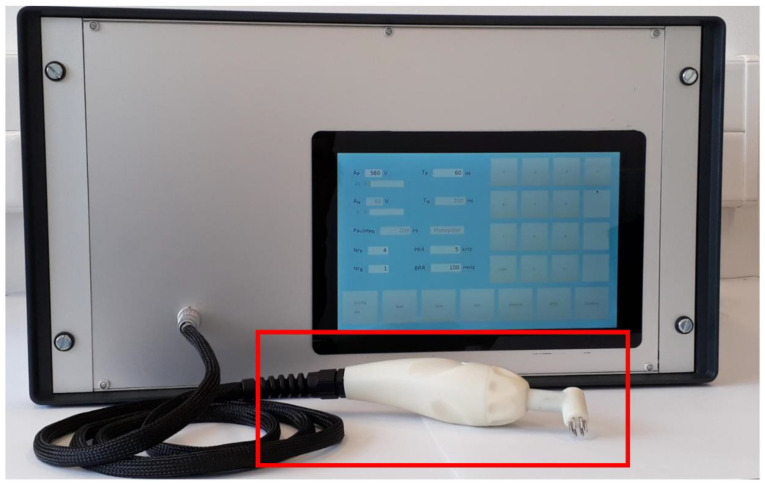
The completed device (pulse generator and applicator) for gene electrotransfer to skin cells. The applicator is shown inside the red frame.

Based on the results of the developed model and the proposed alternative pulse delivery protocol, we designed an applicator with six hexagonal rod electrodes without the central electrode (Figure 1b and red frame in Figure 5). The spacing between adjacent electrodes is 2.5 mm, while the distance between the centers of the opposite electrodes is 9 mm. The electrodes are 10 mm long (outside the housing) with rounded tips and are made of stainless steel 316L. They are intended for multiple use and can be taken off the applicator for easier cleaning and disinfection or for replacement after the determined usage. The geometry of the electrodes allows them to fit different areas of skin on the body, irrespectively of the curvature. The applicator has a built-in green warning light, which informs the operator that the applicator is in contact with the skin and, thus, the device is ready to generate the electrical pulses. In the handle of the applicator there is also a built-in button that is used to trigger the device directly from the applicator. This type of electrode allows for noninvasive pulse delivery with less pain and muscle twitching [29], while also allowing efficient gene electrotransfer.

#### 3.2.2. Device Development

The development of the device started with the design of circuits to power all the other circuits in the device and isolate the low voltage signals at the applicator from the control unit. Then, we designed a circuitry that isolates the high voltage from the control unit (isolation and conversion circuit, A/D and D/A converters). We proceeded with installation of a 30 W high voltage power supply with 21 µF capacitance, which provides a controlled power supply from 0 to 600 V. A switching circuit between the electrodes (electrode commutator) that switches the electrical pulses between the electrodes according to the proposed alternative pulse delivery protocol was also developed. This circuit was connected to the applicator connector. Finally, we added a pulse generator and a current limiter into the housing and developed a graphical user interface displayed on a 10.1″ touchscreen. The completed device (pulse generator and applicator) for GET to skin cells is shown in Figure 5.

The pulse generator is capable of generating square wave electrical pulses from 80 to 600 V with a pulse duration of 10 μs up to 1000 μs at a pulse repetition rate from 0.1 to 5000 Hz.

### 3.3. In Vivo Experiments

**Figure 6:**
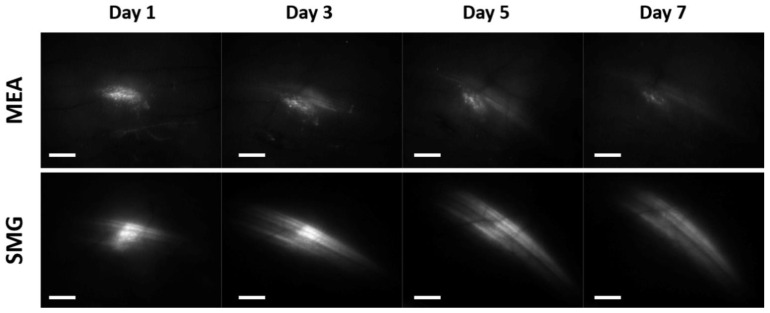
Fluorescence of EGFP protein following GET of the reporter plasmid pEGFP-N1 coding for EGFP in mouse skin. Representative images of EGFP expression in the skin of mice after GET using the multi-electrode array (MEA) electrodes and the previously published pulse delivery protocol: 12 pulses, amplitude 60 V, duration 150 ms, and pulse repetition rate 2.82 Hz ((**upper**) part of the figure); and the newly developed pulse generator in combination with the new applicator and pulse delivery protocol for GET to skin cells—SmartGene (SMG): 18 sequences with 4 pulses, burst repetition rate 50 Hz, amplitude 560 V, duration of each pulse 100 µs, and pulse repletion rate 5 kHz ((**lower**) part of the figure). Scale bar: 2 mm.

To determine the efficacy of the newly developed pulse generator in combination with the new applicator and pulse delivery protocol for GET to skin cells (SmartGene—SMG), they were compared with a previously published pulse delivery protocol for GET to the skin using MEA electrodes and Cliniporator [26,27]. Both pulse delivery protocols successfully transfected mouse skin resulting in detectable EGFP fluorescence already on day 1 after GET, which persisted at least until day 7 after GET (Figure 6).

**Figure 7:**
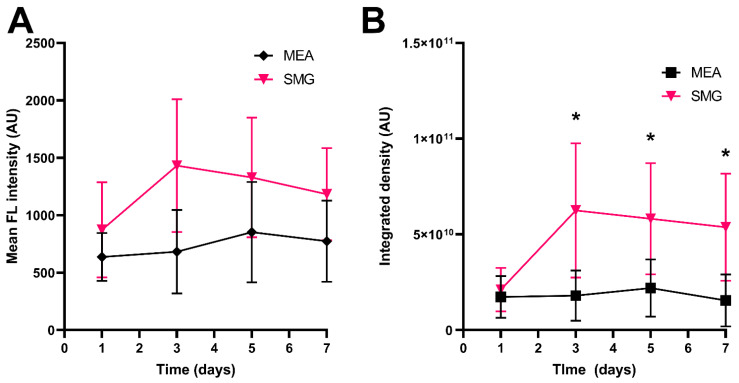
Expression of EGFP protein following GET of the reporter plasmid pEGFP-N1 coding for EGFP in mouse skin. (**A**) Mean fluorescence intensity of EGFP in the transfected skin after GET using the multi-electrode array (MEA) electrodes and the previously published pulse delivery protocol: 12 pulses, amplitude 60 V, duration 150 ms, and pulse repetition rate 2.82 Hz; and the newly developed pulse generator in combination with the new applicator and pulse delivery protocol for GET to skin cells—SmartGene (SMG): 18 sequences with 4 pulses, burst repetition rate 50 Hz, amplitude 560 V, duration of each pulse 100 µs, and pulse repletion rate 5 kHz. (**B**) Integrated density of EGFP in the transfected skin after GET using the MEA electrodes and previously published pulse delivery protocol, and the newly developed pulse generator in combination with the new applicator and pulse delivery protocol for GET to skin cells—SmartGene (SMG). N = 5 (MEA) and N = 4 (SMG). Shown are the mean values ± SD. *—*p* < 0.05, *t*-test. The Shapiro–Wilk test was used to test for normal distribution of the data. A non-parametric *t*-test was performed only for Mean FL intensity (day 3) because a non-normal distribution was found for this point.

The newly developed pulse delivery protocol (SMG) outperformed the MEA pulse delivery protocol resulting in higher mean fluorescence intensity on all the examined days, indicating a higher level of EGFP expression in the transfected area (Figure 7A). Similarly, the SMG pulse delivery protocol showed a statistically significant increase in integrated density on days 3, 5, and 7 after GET, indicating that a larger area of the skin expresses the transfected protein, resulting in more of the transfected protein being produced overall compared to the MEA pulse delivery protocol (Figure 7B).

## 4. Discussion

The aim of our study was to design, develop, and test a new electroporation device (pulse generator and applicator) and a pulse delivery protocol that would maximize gene delivery. The design was based on the target tissue and the effect to be achieved, i.e., gene electrotransfer (GET) of skin cells, while following the previously determined user and technical requirements. We numerically determined the optimal electrode configuration and pulse delivery protocol. We proposed an alternative pulse delivery protocol, which proved to be more successful in achieving a deep and homogeneously reversible electroporated volume, with less damage due to irreversible electroporation than the classical pulse delivery protocol. This also suggests that GET will be more successful with the newly proposed alternative pulse delivery protocol than with the classical pulse delivery protocol. We also focused on the safety of the device and the requirements for clinical use, given the lack of pulse generators for GET that can be used in human studies and in the clinics. Therefore, we designed and developed a new pulse generator, and tested its operation on both a resistive load and in an in vivo gene electrotransfer study.

The results of the performed in vivo study showed that high expression levels of the transfected plasmid DNA proteins can be achieved with the newly developed pulse generator, applicator, and pulse delivery protocol for GET to skin cells in mice. When compared to the previously published pulse delivery protocol for GET to the skin using MEA electrodes [26,27], the newly proposed pulse delivery protocol achieved higher expression levels in the transfected area, as well as higher overall production of the transfected protein.

In developing the pulse generator, we followed the standard EN 60601-1: 2007: Medical electrical equipment—Part 1: General requirements for basic safety and essential performance. This standard is a generally accepted criterion for medical electrical equipment and compliance with this standard has become the main requirement for marketing of medical electrical equipment. Therefore, the pulse generator was tested with a certified and calibrated Fluke ESA620 electrical safety analyzer (Fluke Biomedical, Washington, USA) for medical devices in accordance with the medical standard EN 60601-1: 2007. The electrical safety report showed that the leakage currents are within the allowable leakage currents according to the standard. This means that even in the event of a single fault, the device will not cause harm to the patient. 

However, the device is still not certified as a medical device under the Medical Device Regulation (MDR) 2017/745, although the electrical safety report showed that the device can be used safely. Additional testing by a notified body certified under the current MDR is required to assist us in resolving existing discrepancies, as we were not able to meet all the requirements of the other listed standards and prepare the technical documentation. In addition, we do not have a Quality Management System (QMS) for the procedures and processes required to develop and manufacture a medical device. Therefore, in order to proceed with the development of a clinical electroporator and later with the production, we need to establish QMS and prepare the technical documentation. Overcoming these obstacles will lead to the availability of a certified clinical electroporator for GET to skin cells that can be used with a standardized protocol for new in-human studies.

## 5. Conclusions

This paper presents the design and development of the pulse generator and applicator for gene electrotransfer to skin cells, following user preferences, technical recommendations and treatment protocol. The developed numerical model enabled testing of two different pulse delivery protocols and proposed an alternative pulse delivery protocol, which was then used in vivo for gene electrotransfer to skin cells in mice. The results showed higher mean fluorescence intensity and a statistically significant increase in integrated density after GET with the newly developed pulse generator and applicator for gene electrotransfer to skin cells along with the proposed alternative pulse delivery protocol, compared to the currently used Cliniporator, MEA electrodes, and pulse delivery protocol. However, the device for gene electrotransfer to skin cells and the proposed alternative pulse delivery protocol need further evaluation. In addition, the device needs to be certified as a medical device under the Medical Device Regulation 2017/745 in order to be safely used for new in-human studies.

## Figures and Tables

**Table 1:** Properties of each skin layer used in the numerical model. σ signifies the electrical conductivity; RE—reversible electroporation; IRE—irreversible electroporation.

Skin Layer	Layer Thickness	σ_x_ (S/m)	σ_y_ (S/m)	σ_z_ (S/m)	RE Threshold (V/cm)	IRE Threshold (V/cm)	Maximal σ Increase
Stratum corneum	20 µm	1.10 × 10^−2^	1.10 × 10^−2^	2.23 × 10^−4^	400	1200	100×
Epidermis	0.1 mm	5.82 × 10^−2^	5.82 × 10^−2^	6.36 × 10^−2^	400	1200	3.5×
Papillary dermis *	0.15 mm	7.19 × 10^−2^	7.19 × 10^−2^	7.19 × 10^−2^	300	1200	3.5×
Upper vessel plexus	80 µm	4.22 × 10^−1^	3.86 × 10^−1^	3.86 × 10^−1^	300	1200	3.5×
Supply layer	1 mm	3.12 × 10^−1^	3.12 × 10^−1^	3.19 × 10^−1^	300	1200	3.5×
Deeper vessel plexus	0.1 cm	3.42 × 10^−1^	3.28 × 10^−1^	3.28 × 10^−1^	300	1200	3.5×
Hypodermis *	0.5 cm	6.35 × 10^−2^	6.35 × 10^−2^	6.35 × 10^−2^	300	1200	3.5×
Muscles	2 cm	1.57 × 10^−2^	6.86 × 10^−2^	1.57 × 10^−2^	200 ** 80 **	800	2.5×

* The layers are isotropic and only one value for conductivity is given. ** The threshold value changes according to the direction of the applied electric field with respect to muscle fiber orientation, with the higher value for the perpendicular direction and the lower for the parallel direction.

## Data Availability

The data that support the findings of this study are available within the article.
